# Long-Term Postoperative Pain Prediction Using Higher-Order Singular Value Decomposition of Intraoperative Physiological Responses: Prospective Cohort Study

**DOI:** 10.2196/37104

**Published:** 2022-09-14

**Authors:** Raheleh Baharloo, Jose Principe, Parisa Rashidi, Patrick Tighe

**Affiliations:** 1 University of Florida Gainesville, FL United States

**Keywords:** tensor decomposition, multivariate-temporal decomposition, long-term postoperative pain, higher-order singular value decomposition, SVD

## Abstract

**Background:**

Long-term postoperative pain (POP) and patient responses to pain relief medications are not yet fully understood. Although recent studies have developed an index for the nociception level of patients under general anesthesia based on multiple physiological parameters, it remains unclear whether these parameters correlate with long-term POP outcomes.

**Objective:**

This study aims to extract unbiased and interpretable descriptions of how the dynamics of physiological parameters change over time and across patients in response to surgical procedures and intraoperative medications using a multivariate-temporal analysis. We demonstrated that there is an association (correlation) between the main features of intraoperative physiological responses and long-term POP, which has a predictive value, even without claiming causality.

**Methods:**

We proposed a complex higher-order singular value decomposition method to accurately decompose patients’ physiological responses into multivariate structures evolving over time. We used intraoperative vital signs of 175 patients from a mixed surgical cohort to extract three interconnected, low-dimensional, complex-valued descriptions of patients’ physiological responses: multivariate factors, reflecting subphysiological parameters; temporal factors, reflecting common intrasurgery temporal dynamics; and patients’ factors, describing interpatient changes in physiological responses.

**Results:**

Adoption of the complex higher-order singular value decomposition method allowed us to clarify the dynamic correlation structure included in the intraoperative physiological responses. Instantaneous phases of the complex-valued physiological responses of 242 patients within the subspace of principal descriptors enabled us to discriminate between mild and not-mild (moderate-severe) levels of pain at postoperative days 30 and 90. Following rotation of physiological responses before projection to align with the common multivariate-temporal dynamic, the method achieved an area under curve for postoperative day 30 and 90 outcomes of 0.81 and 0.89 for thoracic surgery, 0.87 and 0.83 for orthopedic surgery, 0.87 and 0.88 for urological surgery, 0.86 and 1 for colorectal surgery, 1 and 1 for transplant surgery, and 0.83 and 0.92 for pancreatic surgery, respectively.

**Conclusions:**

By categorizing patients into different surgical groups, we identified significant surgery-related principal descriptors. Each of them potentially encodes different surgical stimulation. The dynamics of patients’ physiological responses to these surgical events were linked to long-term POP development.

## Introduction

### Background

Persistent pain after acute postoperative pain (POP) occurs in 10% to 50% of patients after common surgical procedures such as cardiac, thoracic, spinal, or orthopedic surgeries [1]. Although even mild levels of persistent POP are associated with decreased physical and social activities [2], 2% to 10% of patients experiencing this type of pain may develop severe levels of pain, delaying their recovery and return to normal daily functioning [3,4]. Furthermore, persistent POP leads to increased direct medical costs through the use of additional resources. Persistent POP appears to be a critical and unrecognized clinical problem [1]. Consequently, the prediction of patients at risk of developing this type of pain, which could inform primary and secondary prevention strategies, has remained inadequate [5].

POP is assumed to stem from various interacting factors including biological, psychological, and social determinants [6]. In different studies [7,8], psychological factors (ie, depression, psychological vulnerability, stress, and catastrophizing) have been suggested as risk factors for the development of persistent POP. Level of education and female sex were seen by some as unlikely to be coupled with persistent POP [7]. However, Holtzman et al [9] identified female sex as a risk factor for developing persistent POP. The relationship between anxiety and the development of persistent POP remains unclear. Although various studies have suggested a significant link between preoperative anxiety and higher levels of persistent POP [10,11], others studies have been unable to replicate this finding. In a systematic review evaluating the association between anxiety and persistent POP in patients undergoing different types of surgery, Hinrichs-Rocker et al [7] found no clear link between the two. In a meta-analysis evaluating 29 research studies, Theunissen et al [12] found that preoperative anxiety was associated with persistent POP in only 55% of the studies.

A frequently replicated finding suggests that the severity of acute POP [1,13,14], especially movement-evoked pain [15-17], is the most striking risk factor significantly associated with persistent POP. Basbaum [18] found that neuroplastic changes in the central nervous system resulting from high intensities of acute POP were a reason for developing persistent pain. In all these studies on the effect of acute POP on the development of persistent POP, a single measurement of acute pain (mean daily value or the worst pain) was examined, and the temporal dynamics of acute pain were discarded. In recent years, the acute POP dynamic (POP trajectory) as a quantification of all apparent and latent factors modulating POP duration and resolution has been examined using different methods to identify abnormal POP resolution [19,20]. Chapman et al [19] approximated daily pain trajectories using a linear mixed model to increase the amount of information extracted from POP recordings. Through this method, 3 pain trajectory patterns were unfolded, yielding new information about the dynamics of POP resolution in a limited time window after surgery. Later, Althaus et al [20] used a latent growth curve on the average pain intensities over the first 5 days after surgery to analyze the mediating effects of POP trajectories within the association between relevant preoperative psychosocial features and chronic POP. Notably, these extensions to pain trajectories generally focused on the daily abstractions of pain intensity ratings and discarded potentially meaningful data pertaining to intraday variations. Furthermore, they used constrained models to approximate the complex dynamic of POP resolution. Baharloo et al [21] extended this line of research by considering POP intensity observations including intraday variations as a time series and used wavelets to approximate the POP temporal dynamics associated with persistent POP.

### Objectives

Although these studies are encouraging, their strategies are inherently limited by a lack of analysis of intraoperative nociception, that is, the sensory nervous system’s response to harmful or potentially harmful stimuli. Pain is a subjective sensory and emotional experience, and every individual may respond differently to a painful stimulus. The characteristics of this response may indicate further development of persistent pain. Hence, we argue that to find a solution to this complex problem, we need to carefully analyze the inherent response to a painful stimulus to characterize the intricate nature of persistent pain. This involves analyzing many parameters, including physiological, emotional, and neuroendocrine parameters. In this study, we considered some of the physiological responses to surgical injury to study how individuals react to a noxious stimulus.

As the autonomic nervous system continuously responds to various surgical stimuli during surgery, vital signs such as heart rate, blood pressure, and respiration can be used as indicators of these responses. During general anesthesia, when a sufficient dose of an anesthetic agent is applied to prevent a response to the skin incision and subsequent surgical trauma, physiological responses induced by surgical stress are not completely attenuated [22]. The sympathetic nervous system inherently changes physiological parameters such as local blood flow, blood pressure, and heart rate in response to noxious stimulation. Anesthetic agents also interfere with this system at different levels. Among the physiological parameters, heart rate and blood pressure may also be modulated by parasympathetic tone [23]. If modeling methods are limited only to physiological parameters, it remains unclear whether any given signal among these multivariate time series results from changes to surgical insults (ie, fluid shifts and nociception from tissue injury) or from the modulation of anesthetic parameters (eg, changes in anesthetic depth). These challenges are compounded by inherent delays in the coupled system owing to the pharmacokinetic and pharmacodynamic principles. Hence, monitoring and analyzing the time series of patients’ physiological responses in relation to a variety of surgical stimuli and nociception imbalance under general anesthesia helps us to indirectly characterize the behavior of the autonomic nervous system in response to nociceptive stimuli, if the temporal dynamics of anesthetic factors can be accounted for alongside similar assessments of the autonomic nervous system. Therefore, such integrated analyses of time series may provide a clue for the development of persistent POP.

Hemodynamic regulation is the result of dynamic interactions between coupled biological systems of different scales and temporal frequencies. Cross-spectral analysis, which determines the relationship between 2 time series as a function of frequency, is a solution for revealing such dynamic interactions in general. However, when the dominant frequencies and scales are unknown or occur over a wide range, it is difficult to use cross-spectral analysis in an exploratory manner. Furthermore, when dealing with nonstationary time series characterized by short and irregularly occurring events, as is the case with intraoperative vital signs, cross-spectral analysis is less descriptive.

This study used the complex higher-order singular value decomposition (HOSVD) method to explore dynamic correlations with lead or lag relations in intraoperative vital signs. The complex-valued vital signs were generated using the original ones and their Hilbert transforms. The key idea was to organize complex-valued vital signs into a third-order tensor with three axes corresponding to individual vital signs (physiological parameters), time during surgery, and patients. We then fit the HOSVD to identify a set of low-dimensional complex-valued factors (features) that capture variability along each of these 3 axes.

Complex HOSVD identifies separate low-dimensional complex-valued factors, each of which corresponds to subphysiological parameters with common within-surgery dynamics and variable across-patient dynamics. We then investigated how surgical mechanisms in different procedures emerged as the patients’ physiological responses occurred. The investigation elucidated how the particular dynamics of each surgical service were captured in individual factors, which had different characteristics. We discuss how complex HOSVD can extract descriptors of physiological responses in which individual factors potentially correspond with interpretable activities such as tidal volume determination and autonomic regulation during surgery.

**Figure 1:**
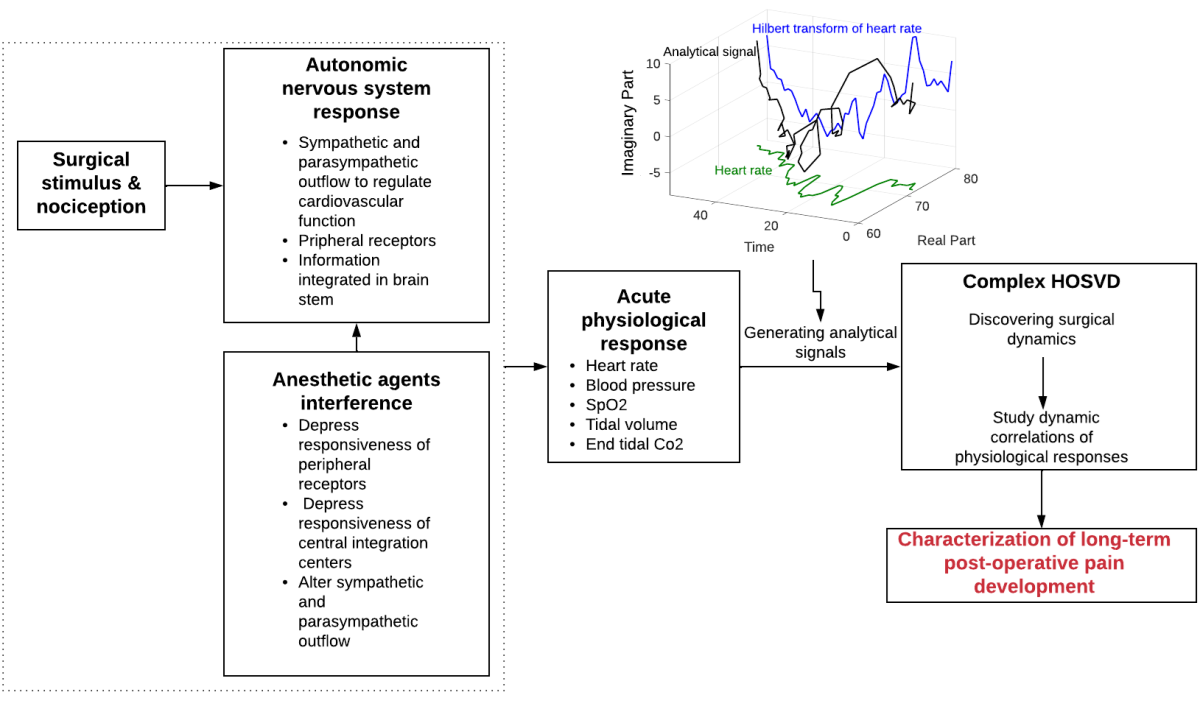
Flow diagram of the work proposed in this study. Multivariate intraoperative vital signs as indicators of the dynamic interplay among the surgical stimulus, autonomic nervous system, and anesthetic agents are analyzed through tensor decomposition to characterize long-term postoperative pain.

Finally, we used the complex-valued factors as new bases to describe physiological responses. After projection onto the subspace, the complex correlations between each intraoperative time series and the complex-valued factors were manifested in the magnitudes and phases of the correlations. We used the phases of the correlations to predict *mild* versus *moderate-severe* levels of pain on postoperative days 30 and 90. We demonstrated that the dissimilarities between these 2 pain categories were relatively expressed in the phase information of the physiological responses with respect to surgical dynamics. Figure 1 illustrates the relationship between the proposed tasks and the underlying biological subsystems.

## Methods

### Discovering Surgical Multivariate-Temporal Dynamics Through Complex HOSVD

#### Intraoperative Vital Sign Recording

During surgery, patients experience various disturbances with respect to the normal activities of different body systems. Therefore, it is essential to monitor a patient’s physiological status. Physiological monitoring systems can continuously measure and monitor various vital signs using electrodes and sensors connected to the patient. Routinely measured vital signs may include electrical activity of the heart (through an ECG), heart rate, respiratory rate, blood pressure, cardiac output, body temperature, peripheral capillary oxygen saturation (SpO_2_), end-tidal carbon dioxide (EtCO_2_), and exhaled tidal volume.

*Blood pressure* is the pressure generated by circulating blood on the walls of blood vessels and usually points to the pressure in the large arteries. Blood pressure is commonly expressed as systolic and diastolic pressures. Systolic pressure refers to the amount of pressure in the arteries when the heart contracts to pump blood into circulation. Diastolic pressure refers to the pressure when the heart relaxes after contraction. During surgery, blood pressure can be measured using invasive and noninvasive methods. Invasive monitoring of blood pressure involves direct estimation of arterial pressure by inserting a cannula into an appropriate artery. This provides continuous beat-by-beat monitoring of the patient’s blood pressure. Noninvasive monitoring uses an oscillometric technique with an automated cuff.

*SpO_2_* is measured noninvasively using a pulse oximeter to provide an approximation of the arterial hemoglobin oxygen saturation. A sensor is clipped over the finger, and the pulse oximeter continuously emits and absorbs a light wave passing through the capillaries. As the oxygen binding of hemoglobin causes changes in the color of blood, variations in the red and infrared light absorption spectra in the arterial phase provide an estimate of the oxygen content within the arterial system. The pulse oximeter also provides heart rate in beats per minute, with an average rate of over 5 to 20 seconds.

*EtCO_2_* represents the amount of carbon dioxide in exhaled gas. Tidal volume represents the volume of air displaced between inhalation and exhalation. These 2 parameters can be used to assess ventilation.

Isoflurane and sevoflurane (both included in the same class of medicines) were used as inhaled anesthetic agents in this study, both of which are known to have a depressive effect on the autonomic nervous system. The end-tidal concentration of inhalational anesthetic gases, such as isoflurane and desflurane, is related to the alveolar concentration of the anesthetic, which in turn is related to the concentration of anesthetic gas at the target effect site (eg, the central nervous system). End-tidal concentrations of anesthetic gas are measured in real time throughout anesthesia as an indicator of the depth of anesthesia. Increasing amounts of anesthesia lead to amnesia, hypnosis, muscle relaxation, and eventual suppression of sympathetic responses to noxious stimuli such as incision. Anesthetic management often requires a balance between the amount of anesthetic delivered and the degree of noxious stimuli, which is further modulated by interindividual differences in anesthetic sensitivity.

It is commonly accepted that the degree of noxious stimuli observed during surgery denotes the degree of tissue injury, which is also related to POP intensity. No direct measures of noxious stimuli are available in clinical practice. However, by considering the amount of anesthetic administered following incision and the physiological variabilities observed in this period in the presence of a given amount of anesthesia, we can deduce the overall relationship between nociceptive-triggered sympathetic stimulation and anesthesia-induced sympathetic suppression. Without considering indicators of both physiological and anesthetic states simultaneously, it remains difficult to ascribe any given change in one of these dimensions to a third entity such as surgical nociception.

In this study, we used eight vital parameters—heart rate, heart rate-SpO_2_, SpO_2_, systolic blood pressure, diastolic blood pressure, EtCO_2_, tidal volume exhaled, and end-tidal concentration of isoflurane and sevoflurane—as superficial and imperfect indicators of autonomic nervous system activity or state. These parameters were subjected to tensor decomposition analysis to characterize long-term POP.

*Long-term POP* was defined as a self-reported mean value of pain on postoperative day 30 using a numeric rating scale (0=no pain and 10=worst pain). Although this method is not an ideal assessment of pain, and its potential subjective bias makes it less reliable, different studies have reported a significant correlation between this method and the pain measured by different candidate technologies such as physiological parameters or cerebral hemodynamic changes for pain assessments [24].

#### Application of Singular Value Decomposition to Large-Scale Intraoperative Data Analysis

Before describing complex-HOSVD in our analysis, we first discuss the potential application of singular value decomposition (SVD) to large-scale intraoperative data analysis. Consider a recording of I_1_ intraoperative vital signs over I_3_ different patients. We assume that vital signs are recorded at I_2_ time points for each patient, but recordings of variable duration can be cut to a common window of time to fit in with this constraint. The collection of these series is naturally represented as an *I*_1_×*I*_2_×*I*_3_ array of vital signs and a third-order tensor such as 
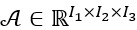
. Each member of this tensor, *a_i_*1*i*2*i*3, denotes the recorded value of each vital sign *i*_1_ at time point *i*_2_ for patient *i*_3_.

Given that patients experience various surgical stimuli, such large multiway arrays (tensors) are challenging to analyze and interpret, particularly when recordings are performed over a wide range of surgical services. Physiological responses to identical surgical stimuli exhibit significant interpatient variability.

Under the assumption of having the intraoperative vital signs recorded for just 1 patient, we obtained a matrix, **A***_I_*_1_*_I_*_2_, which holds the values for each vital sign *i*_1_ and time point *i*_2_. Such a matrix is difficult to interpret when different vital signs with distinct temporal dynamics are involved in experiments.

**Figure 2:**
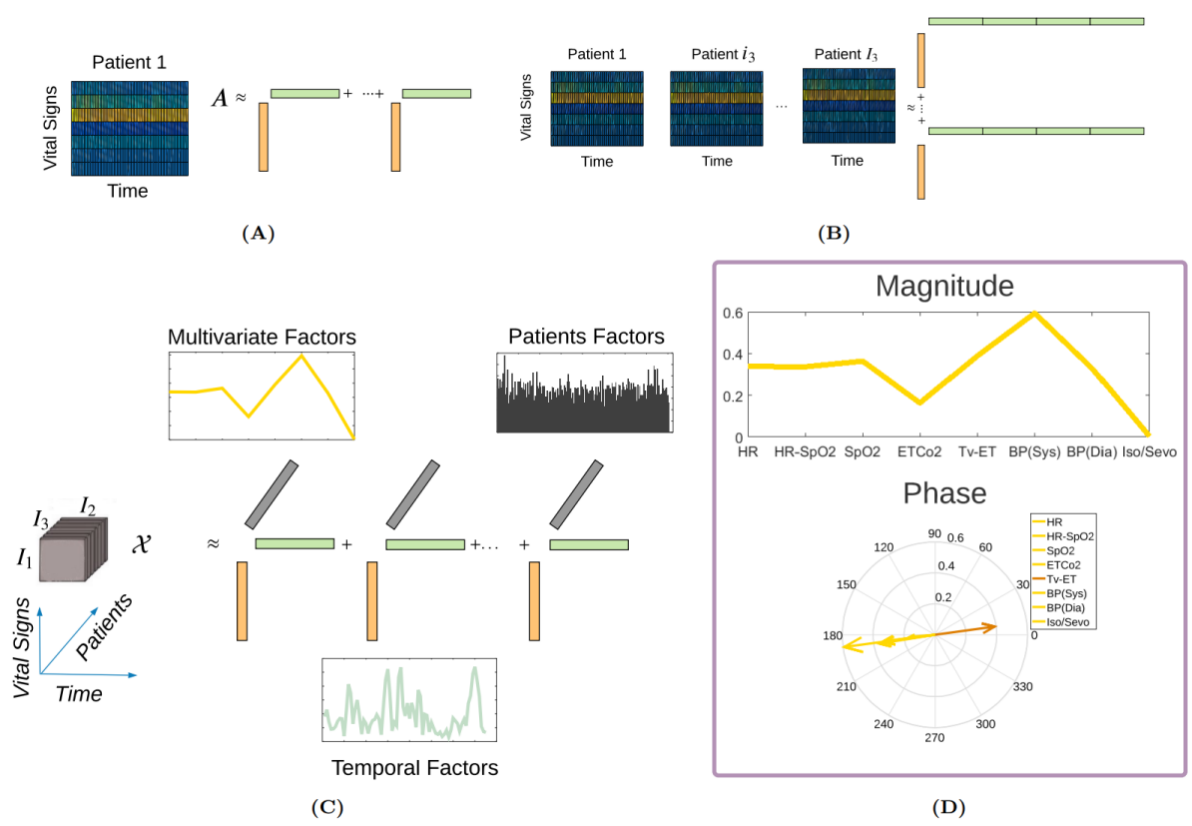
Shaping data matrices/tensors for decomposition. (A,B) Singular value decomposition summarizes matrix A by carrying out a decomposition into R number of ranked-one matrices. (C) Decomposition of third-order tensor X provides prototypical pattern across intraoperative vital signs (multivariate factors), temporal dynamic across time (temporal factors) and patients-specific variations (patients factors). (D) Illustration of a single multivariate factor plotted with respect to magnitude and phase.

SVD summarizes this matrix by carrying out a decomposition into R number of ranked-one matrices (components), such as in equation 1, to approximate the original data matrix.



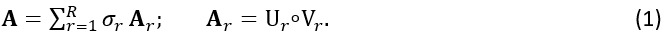



where ° denotes the outer product of the vectors. This decomposition provides a low-dimensional subspace (a new coordinate system) with R dimensions to describe the original high-dimensional data with I_1_ or I_2_ dimensions. Whenever decomposition is applied, we use the terms dimensions, components, and ranked-one matrix or tensor interchangeably, but they convey the same meaning. Each ranked-one matrix, indexed by r, holds a coefficient across vital signs, *u_ri_*_1_, and a coefficient across points in time *v_ri_*_2_. These vectors represent the multivariate-temporal dynamics discovered in the original data matrix. In this paper, we call the vectors *U_r_* temporal modes (factors; yellow and green vectors in Figure 2A). Each coefficient (element) of the multivariate (or temporal) mode vectors contains 2 important pieces of information. The absolute value of the coefficient provides a measure of the contribution of a particular vital sign (or time point) for that mode. If the coefficient is complex valued (as is the case with this study), the angle defined by the real and imaginary parts provides an explanation of the phase of that coefficient (element) in relation to the others vibrating at the frequency associated with that particular mode [25]. To account for the variability of vital signs among patients and to simplify data tensor A, one approach is to concatenate multiple data matrices such as **A***_I_*_1_*_I_*_2_ (one for each patient), thereby converting the data tensor into an *I*_1_×*I*_2_*I*_3_ matrix and then applying SVD to this matrix (Figure 2B). In this way, the R temporal modes are of length I_2_I_3_ and do not capture the common temporal dynamics across patients.

In this study, we performed decomposition directly on the original data tensor A (Figure 2C) rather than transforming it into a matrix. The HOSVD method then provides the following decomposition:



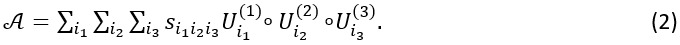



Analogous to SVD, we can think of *U*^(1)^ as a prototypical pattern across intraoperative vital signs and *U*^(2)^as a temporal dynamic across time. These multivariate and temporal modes represent dynamics that are common among patients. The third set of modes, *U*^(3)^, *patient factors* (Figure 2C), represents patient-speciﬁc variations in the multivariate-temporal dynamics identiﬁed by the method.

Furthermore, to capture the propagating dynamics, the real-valued vital signs are augmented with their Hilbert transforms to form a complex-valued third-order tensor such as 
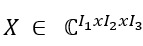
. The HOSVD decomposition in equation 2 also holds for the complex-valued tensor *X* [26]. The complex-HOSVD identiﬁes dynamic factors that carry additional information related to phase. Figure 2D illustrates a single multivariate factor plotted with respect to magnitude and phase, where each element of the multivariate factor represents a particular vital sign recorded during surgery. The phase was plotted between 0 and 2π, representing the relative phases of the elements. The phase plotted on a circular grid exhibited an interesting feature. All elements of the multivariate factor showed the same phase, except for the element associated with the contribution of tidal volume. The tidal volume is selected by the anesthesiologist during surgery, but it still influences the heart rate and blood pressure in patients.

### Ethics Approval

This prospective cohort study was approved by the University of Florida Institutional Review Board-01 (IRB #201500153) as the National Institutes of Health–funded the Temporal Postoperative Pain Signatures (TEMPOS) protocol.

## Results

### Experimental Setup and Discovered Surgical Dynamics

#### Overview

In this study, we designed and tested a complex-HOSVD–based metric projection to characterize patients’ physiological dynamics from intraoperative vital signs collected during surgery at a rate of 1 sample per minute for at least 75 minutes. The intraoperative vital signs were augmented by the Hilbert transform [27] to create complex vital signs. Here, a 2D tensor (matrix) represents the time-varying dynamics of the different intraoperative vital signs for each patient. The 175 second-order tensors constructed from the intraoperative vital signs of 175 patients undergoing a relatively wide range of surgical procedures, including orthopedic, urology, colorectal, transplant, pancreatic and biliary, and thoracic procedures, were stacked on top of each other to generate a 3D tensor to discover mixed surgical dynamics. The complex principal multivariate-temporal factors extracted through complex HOSVD were compared with the multivariate-temporal dynamics extracted after grouping the patients based on their surgical service. The complex-HOSVD decomposition resulted in an approximation of the multivariate factor and temporal factors with sizes of 8×4 and 75×32, respectively. Thus, a total of 128 (F=4×32) features were extracted from 600 features of the tensors.

**Figure 3:**
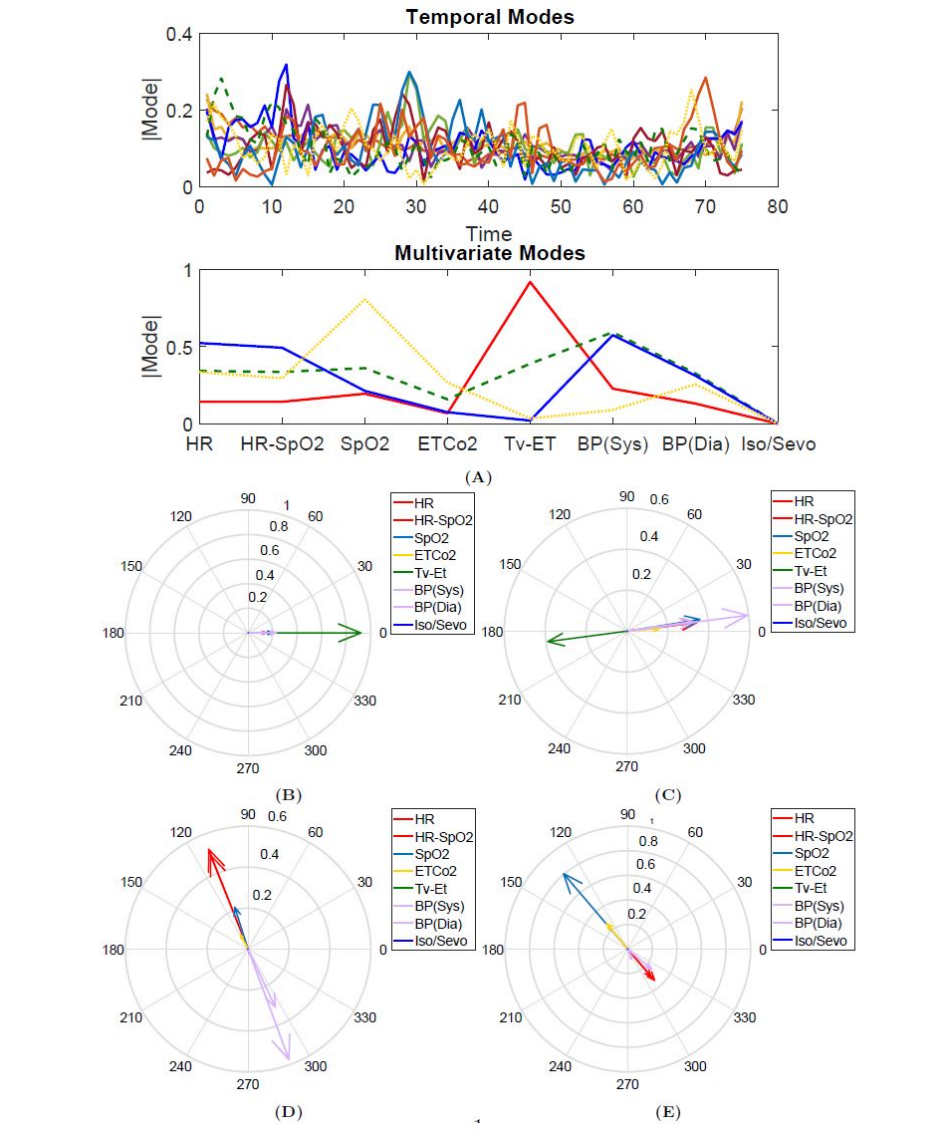
Illustration of the multivariate and temporal factors extracted through complex higher-order singular value decomposition (HOSVD). (A) The outer product of the temporal and multivariate factors generates the contributing components in the decomposition of X. For intraoperative vital signs, each of the elements of a multivariate mode represents a particular vital sign. The magnitude and phase of the element explain how the vital signs are related to each other within that factor. The phase of each element describes the relative phase of the vital sign’s vibration relative to the other vital signs for that multivariate factor. This depiction allows for interpretation of the complex HOSVD output for intraoperative vital signs. Each multivariate factor identifies the vital signs involved in that pattern of physiological response in addition to the relative phase of that vital sign’s activation time. (B, C, D, E) The phase portraits associated with the multivariate factors are shown in red, blue, green, and yellow.

The complex-HOSVD characterized surgical dynamics over a wide range of surgical services (refer to Table 1 for the list of surgical services). Remarkably, complex-HOSVD extracted only 4 multivariate factors to capture within-patient and across-patient intraoperative dynamics. The temporal evolution of these multivariate factors was captured by 32 temporal factors and showed substantially different characteristics potentially affected by surgical services. The multivariate-temporal factors of the complex-HOSVD are shown in Figure 3. The first multivariate factor (red in Figure 3A) indicated a strong contribution of tidal volume for this factor. As stated before , the anesthesiologist selects this variable during surgery, and it can impact the patient’s hemodynamic response by changing the venous return to the heart, heart rate, and cardiac output. The corresponding elements of this mode oscillated in the same phase (Figure 3B). The second multivariate mode (blue in Figure 3A) emphasizes the contribution of heart rate and blood pressure for this factor. According to its phase plot (Figure 3C), all elements of this factor had the same phase, except for the element associated with the participation of tidal volume. The third multivariate mode (green in Figure 3) indicates a slightly different elemental participation from that indicated by the second factor. The phase information for this multivariate mode indicates a phase difference between blood pressure and the other elements of this mode (Figure 3D). Finally, the fourth multivariate mode (yellow in Figure 3A) highlights the contribution of SpO_2_ and EtCO_2_ while also revealing the phase difference between these two and the other contributing elements for this mode (Figure 3E).

**Table 1:** List of studied surgical services and number of patients in each pain group, in each case.

Surgery	Total number of patients, n	Number of patients in pain group, n (%)	
		Mild	Moderate-severe
Thoracic	37	24 (65)	13 (35)
Orthopedics	35	22 (63)	13 (37)
Urology	60	52 (87)	8 (13)
Colorectal	65	51 (78)	14 (22)
Transplant	11	8 (73)	3 (27)
Pancreas and biliary	34	25 (74)	9 (26)

#### Grouping Patients Based on Surgical Service and Discovery of Surgery-Related Features

Different surgical procedures lead to different patterns of tissue injury. Hence, the type of procedure specifies the organ, organ system, or tissue involved as well as the degree of invasiveness. The influence of the type of surgery on the development of chronic POP is well established. Longer and more complicated operations, as well as those associated with neuropathic patterns of POP, are often linked with a higher risk of chronic pain development, although the pattern is irregular and also related to the type of tissue involved in the surgery. In our analysis, the evolutionary dynamics of intraoperative vital signs had a temporal factor that was significantly affected by the type of surgery. Therefore, in this section, we divided the patients into subgroups related to different surgical procedures and investigated surgery-related features associated with the development of long-term POP. Surgery-related features may correspond to a power increase or decrease distributed over multiple intraoperative vital signs as well as changes in the activation of oscillating frequencies of multivariate factors expressed by temporal modes. In this study, the input to the complex HOSVD algorithm was built from the time-varying contents of 7 intraoperative vital signs with a length of 50 minutes (starting 10 minutes before incision time during surgery). The peri-incisional period was selected in an effort to standardize the phase of surgery as well as to account for potential differences in POP in short- duration procedures versus long-duration procedures. Data were extracted from the Epic electronic health record system by Epic Systems Corporation, which contains an anesthetic information management module. We increased the number of patients in each subgroup by decreasing the length of intraoperative vital signs. We divided 242 patients into 6 groups based on the surgical services they received. The surgical groups included thoracic, orthopedic, urological, colorectal, transplant, and pancreatic and biliary surgeries. The surgical services and surgeries used in this study and the number of patients in each surgery group are summarized in Table 1.

**Figure 4:**
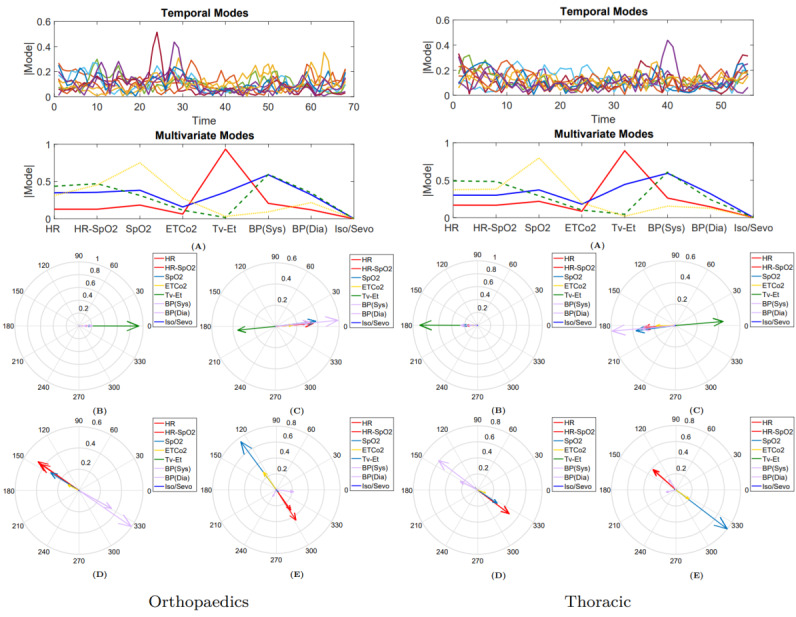
Comparison of the multivariate and temporal factors extracted through complex higher-order singular value decomposition (HOSVD) for 2 different surgical services. (A) The outer product of the temporal and multivariate factors generates the contributing components in the decomposition of X. For intraoperative vital signs, each of the elements of a multivariate mode represents a particular vital sign. The magnitude and phase of the element explain how the vital signs are related to each other within that factor. The phase of each element describes the relative phase of the vital sign’s vibration relative to the other vital signs for that multivariate factor. This depiction allows for interpretation of the complex HOSVD output for intraoperative vital signs. Each multivariate factor identifies the vital signs involved in that pattern of physiological response in addition to the relative phase of that vital sign’s activation time. (B, C, D, and E) In orthopedic and thoracic surgeries, the phase portrait associated with the multivariate factors is shown in red, blue, green, and yellow.

We attempted to identify how surgery-specific mechanisms are associated with patients’ physiological responses over the course of the procedure. Here, we showed that complex-HOSVD could characterize surgery-related dynamics using the physiological responses of a group of patients who underwent the same surgical procedure. Figure 4 illustrates the surgical dynamics characterized by complex-HOSVD for two types of surgery (orthopedic and thoracic). Once again, complex HOSVD summarized both the within-patient physiological responses and the across-patient dynamics in quite a few multivariate factors. These factors offer slightly different elemental contributions while exhibiting the same relative phase portrait. The time course of these factors was substantially different for the different types of surgeries. In essence, the multivariate factors indirectly encoded the sympathetic activities to compensate for variations in hemodynamic parameters (eg, autonomic regulation) under general anesthesia, and these signatures were modulated by the physiological state during surgery (captured by temporal factors).

### Physiological Responses During Surgery and POP

#### Overview

The complex principal multivariate-temporal factors extracted through complex HOSVD were used as new bases to describe the correlations of physiological dynamics and to gain insight into any lead or lag relations among individual responses expressed in instantaneous phases of the complex vital signs.

We divided 242 patients (mean age 62 years, SD 8 years), of which 128 (52.9%) participants were women, into 2 groups based on verbal evaluation of average pain on days 30 and 90 after surgeries including orthopedics, thoracic, urology, colorectal, transplant, and pancreatic biliary surgeries. Patients reporting an average pain intensity of ≤3 were categorized as *mild*. Patients reporting an average pain intensity >3 were considered *not-mild* or *moderate-severe*. This distinction is clinically relevant, as moderate to severe pain ratings generally require analgesic interventions [28]. The number of patients in each group is reported in Table 1.

The subspace provided by complex HOSVD can be used directly in a classification task. However, the corresponding bases do not contain any category information that is functional in modeling the dissimilarity among the categories of data. To obtain the most salient multivariate-temporal factors for this classification task, we used a rank feature method based on the Fisher ranking, and the top 3 components were selected. The projection was performed on a 3D data manifold, which in our study was the top 3 dimensions that provided the highest Fisher scores. The phase information of the projected data points was used to classify *mild* versus *moderate-severe* classes on postoperative days 30 and 90 through linear discriminant analysis (LDA).

#### Results for Postoperative Day 30

**Figure 5:**
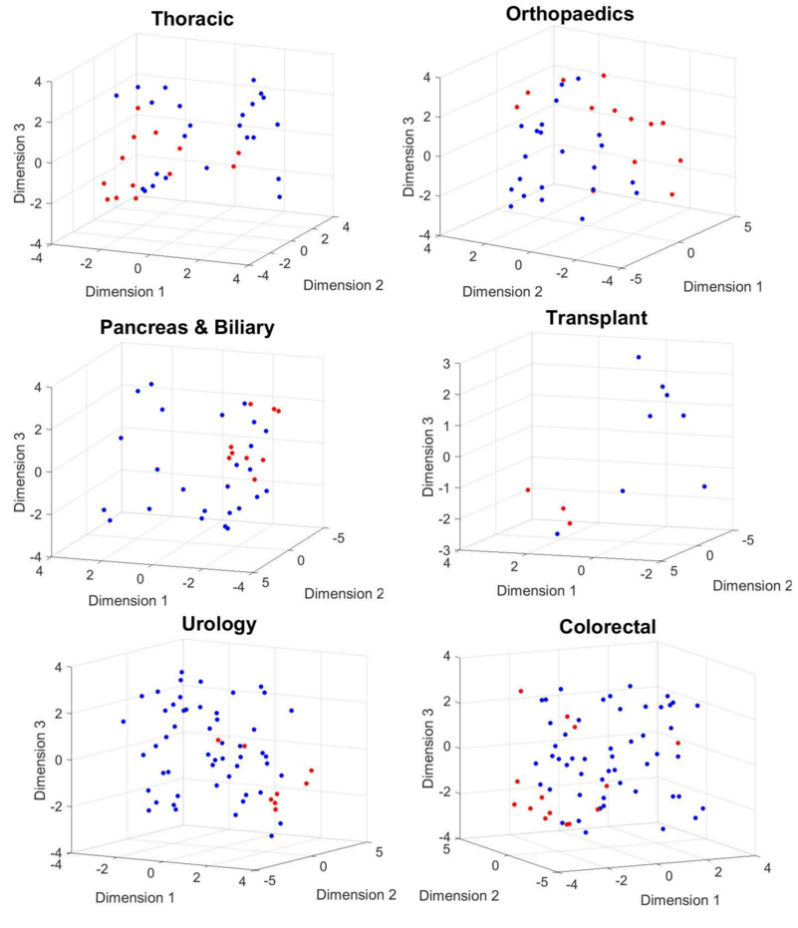
The phase information of the projected data points onto a 3D manifold extracted using complex higher-order singular value decomposition. Mild (blue dots) versus moderate-severe (red dots) levels of pain on day 30 after surgery are considered for this plot.

**Figure 6:**
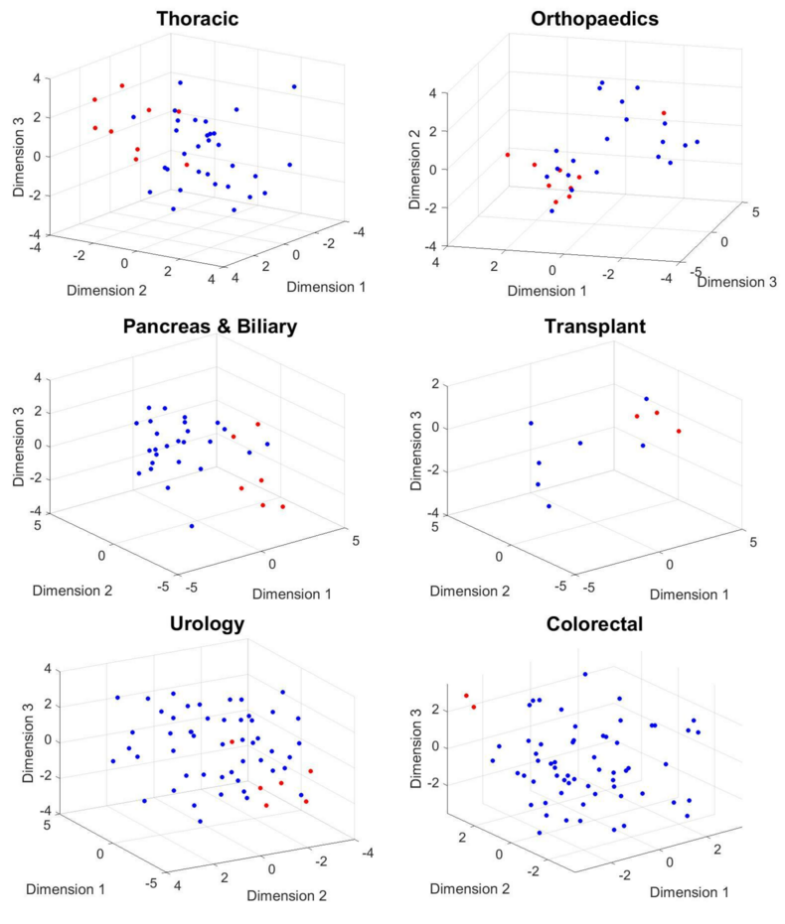
The phase information of the projected data points onto a 3D manifold extracted using complex higher-order singular value decomposition. Mild (blue dots) versus moderate-severe (red dots) levels of pain on day 90 after surgery are considered for this plot.

We investigated the performance of LDA using a 5-fold cross-validation procedure. The method achieved a true positive rate (TPR) and positive predictive value (PPV) of 0.69 and 0.60 for thoracic surgery, 0.77 and 0.67 for orthopedic surgery, 1 and 0.75 for transplant surgery, and 0.63 and 0.71 for urological surgery, respectively. In contrast, the PPV and TPR for the *moderate-severe* class was 0.44 and 0.57 in pancreatic surgery and 0.43 and 0.86 in colorectal surgery, respectively. The results are presented in Table 2. Figures 5 and 6 show the scatter plot of the phase information for patients projected onto the 3D subspace for different surgical groups. Patients with moderate-severe pain on postoperative day 30 were almost well clustered in the thoracic, orthopedic, transplant, and colorectal surgical groups. This finding indicates that the dynamics of patients’ physiological responses to surgical stimulation are linked to long-term POP development. Many patients in the same pain category responded to surgical stimulation, with a small band of variation in their phases. This phenomenon was captured even better for moderate-severe levels of pain on postoperative day 90 (Figure 6).

**Table 2:** Performance of LDA^a^ to discriminate moderate-severe versus mild pain categories for postoperative day 30 without rotation. The phase information of the projected data points on a 3D manifold was used in the experiments. The patients were categorized based on their surgical services

Surgery	Confusion matrix (patients)					Precision (PPV^b^)		Sensitivity (TPR^c^)		Specificity (TNR^d^)		AUC^e^
	TP^f^	FP^g^	FN^h^	TN^i^								
Thoracic	9	6	4	18	0.60		0.69		0.75		0.78	
Orthopedics	10	5	3	17	0.67		0.77		0.77		0.80	
Urology	5	2	3	50	0.71		0.63		0.96		0.87	
Colorectal	6	1	8	50	0.86		0.43		0.98		0.75	
Transplant	3	1	0	7	0.75		1		0.88		0.92	
Pancreas and biliary	4	3	5	22	0.57		0.44		0.88		0.80	

^a^LDA: linear discriminant analysis.

^b^PPV: positive predictive value.

^c^TPR: true positive rate.

^d^TNR: true negative rate.

^e^AUC: area under curve.

^f^TP: true positive.

^g^FP: false positive.

^h^FN: false negative.

^i^TN: true negative.

#### Results for Postoperative Day 90

Given that healing times vary between procedures, and the International Classification of Diseases defines persistent POP as lasting for at least 3 months after surgery, we repeated the exact set of experiments to classify patients who reported *mild* versus *moderate-severe* levels of pain on postoperative day 90. We observed that although the number of patients included in the *moderate-severe* class decreased for all surgical groups, we achieved almost the same or higher performances in detecting patients who developed *moderate-severe* versus *mild* levels of pain (except for urological and orthopedic surgeries). The results are summarized in Table 3.

**Figure 7:**
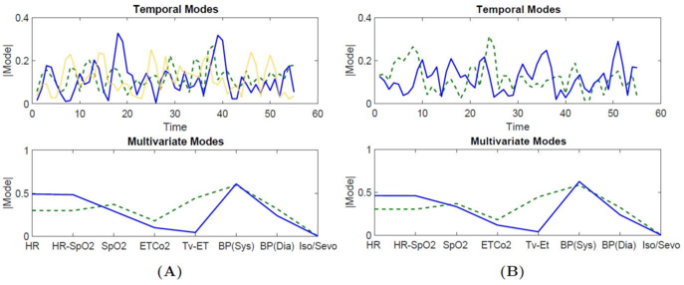
The contributing multivariate-temporal factors (blue, green, and yellow indicate rank from the highest to the lowest) for thoracic surgery. (A) Postoperative day 30: The multivariate modes show that the most dissimilarities between mild and moderate-severe levels of pain are encoded in variations of heart rate and blood pressure (blue and green vectors). Time evolution of the multivariate modes are encoded in temporal modes. (B) Postoperative day 90: The two multivariate factors (blue and green vectors) emphasize the strong contribution of heart rate and blood pressure while they have distinct phase portraits.

Figure 7 compares the contributing multivariate-temporal factors for the first 3 leading components with the highest Fisher scores, differentiating between *mild* and *moderate-severe* levels of pain on postoperative days 30 and 90 for thoracic surgery. For postoperative day 30, the first and second multivariate factors emphasized the roles of heart rate and blood pressure. The activation of the second multivariate mode (green vector) was captured within two distinct temporal factors (green and yellow vectors). Figure 5 illustrates that almost all patients who developed moderate-severe levels of pain on postoperative day 30 had negative phases with respect to the first and second dimensions, mostly focusing on changes in heart rate and blood pressure. For postoperative day 90, the first and second multivariate factors emphasized the roles of heart rate and blood pressure. The activation of the first multivariate mode (blue vector) was captured within two distinct temporal factors (blue and green vectors). Figure 6 illustrates that almost all patients who developed moderate-severe levels of pain on postoperative day 90 had positive phases with respect to the first and third dimensions and negative phases with respect to the second dimension.

**Table 3:** Performance of LDA^a^ to discriminate moderate-severe versus mild pain categories for postoperative day 90 without rotation. The phase information of the projected data points on a 3D manifold was used in the experiments. The patients were categorized based on their surgical services.

Surgery	Confusion matrix (patients)					Precision (PPV^b^)		Sensitivity (TPR^c^)		Specificity (TNR^d^)	AUC^e^
	TP^f^	FP^g^	FN^h^	TN^i^							
Thoracic	6	2	3	29	0.75		0.67		0.94		0.87
Orthopedics	6	5	3	15	0.55		0.67		0.75		0.73
Urology	2	1	4	49	0.67		0.33		0.98		0.88
Colorectal	2	0	0	60	1		1		1		1
Transplant	2	1	1	6	0.67		0.67		0.86		0.90
Pancreas and biliary	4	2	2	24	0.67		0.67		0.92		0.92

^a^LDA: linear discriminant analysis.

^b^PPV: positive predictive value.

^c^TPR: true positive rate.

^d^TNR: true negative rate.

^e^AUC: area under curve.

^f^TP: true positive.

^g^FP: false positive.

^h^FN: false negative.

^i^TN: true negative.

### Rotating the Physiological Responses With Respect to Patients’ Dynamic Variation

#### Overview

As discussed earlier, each complex HOSVD component identifies subphysiological parameters (multivariate factor) with common intrasurgery temporal dynamics (temporal factor), which were differentially activated across patients. Overall, the complex HOSVD model uncovered a reasonable portrait of surgical dynamics (population dynamics), in which distinct subsets of physiological parameters were active at different times during surgery and whose variation across patients was encoded in individual dynamic variables. Until now, we have used the common multivariate-temporal dynamics as new bases to describe physiological responses; hence, we discarded individual dynamic variations encoded in *patient factors.* However, for a better representation of the dynamics, it is essential to associate each principal component (as one base of the subspace) with each dynamic mode of the patients’ physiological responses. The coordinate systems provided by the common multivariate-temporal factors and the patients’ multivariate-temporal dynamics are not necessarily the same (ie, not aligned) [29]. Given that all factors extracted through complex HOSVD are complex-valued factors, the patient-specific variations for the multivariate-temporal dynamics identified by the method contain scaling and rotational adjustments that appear in the outer product of the multivariate-temporal dynamics with the patients’ factors.

**Figure 8:**
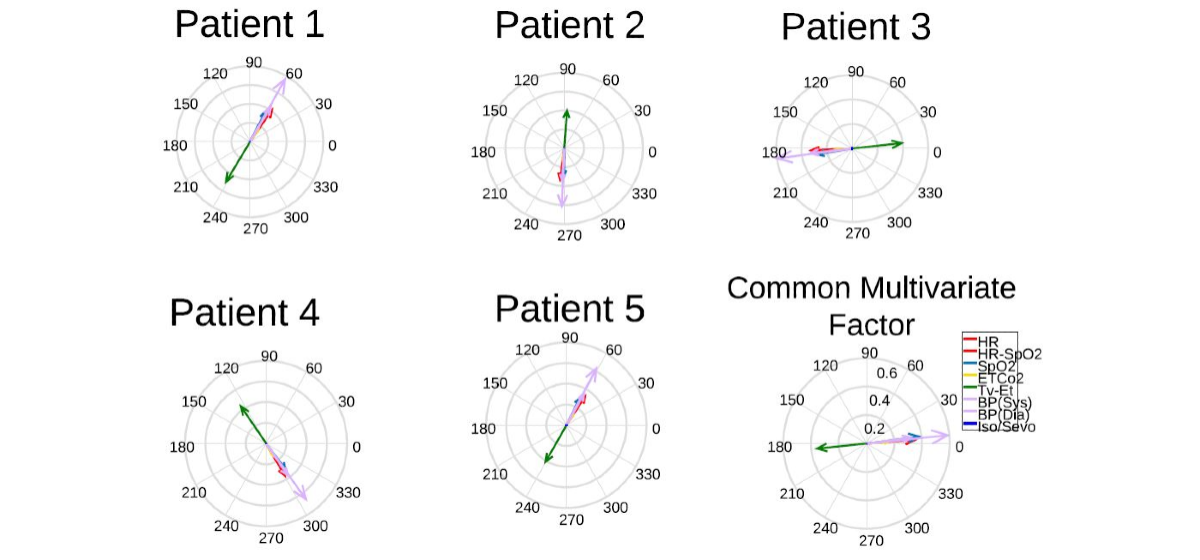
The multivariate dynamic variation for different patients in transplant surgery. For intraoperative vital signs, each of the elements of a multivariate mode represents a particular vital sign. The magnitude and phase of the element explain how the vital signs are related to each other within that factor. The phase of each element describes the relative phase of the vital sign’s vibration relative to the other vital signs for that multivariate factor. Although the elements of the multivariate factors for different patients have the same relative phase, the dynamics are not exactly aligned.

Figure 8 illustrates multivariate dynamic changes across 5 patients in transplant surgery. For simplicity, temporal factors are discarded in this figure, but the same adjustments apply for temporal factors as well.

**Figure 9:**
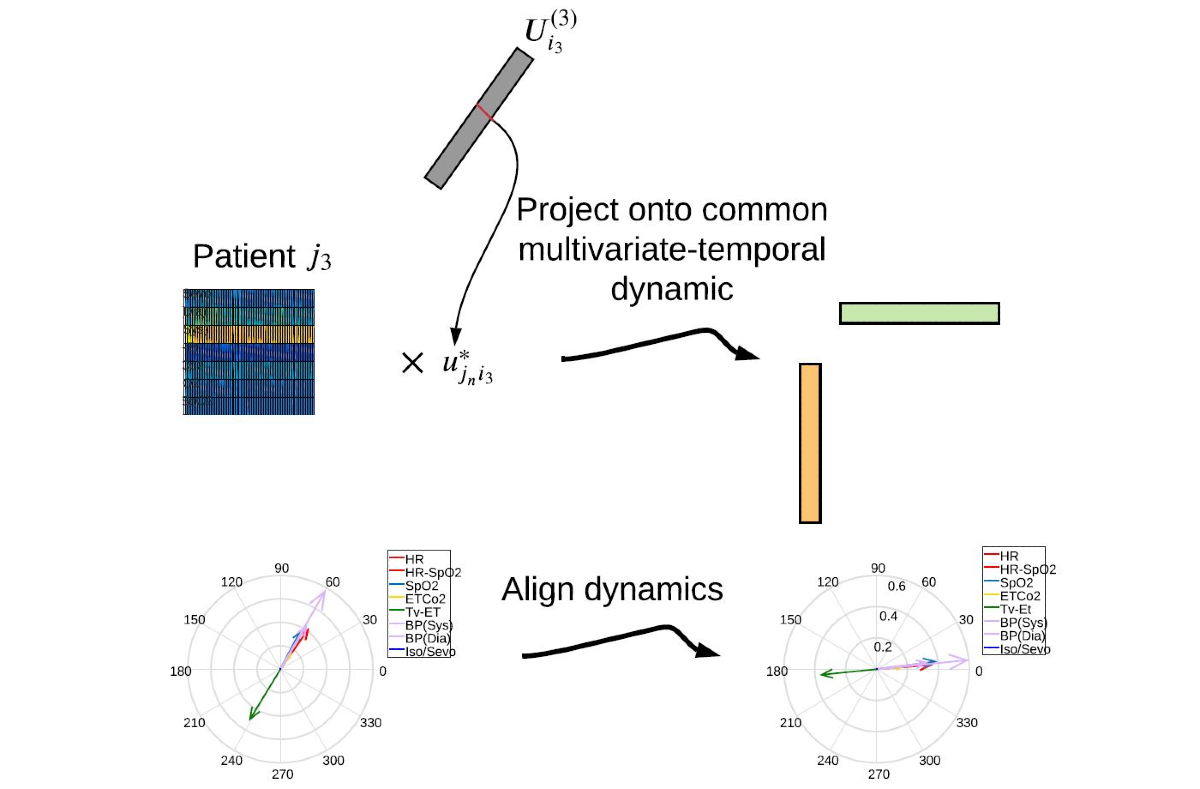
Rotation of physiological responses before projection to align with the common multivariate-temporal dynamic. For simplicity, one component is shown here.

To compare the complex correlations between each physiological response and the extracted multivariate-temporal dynamics, it was essential to have a common coordinate system for all patients. Simultaneously, to account for the dynamic variation across patients, instead of rotating the dynamics, the complex conjugate of elements given by the patients’ factors may be used to scale and rotate the physiological responses before projection onto the subspace. The process can be performed separately for each complex HOSVD component. From a geometric point of view, the process can be considered an active transformation in which the position of a point changes in a coordinate system, whereas a passive transformation changes the coordinate system in which the point is described. Figure 9 illustrates how the process works.

Once the new projections were obtained, we repeated the same set of experiments to explore the dynamic correlations in intraoperative vital signs. Again, the phase information of the projected data points was used to classify *mild* versus *moderate-severe* classes on postoperative days 30 and 90 through LDA.

#### Results for Postoperative Day 30

**Figure 10:**
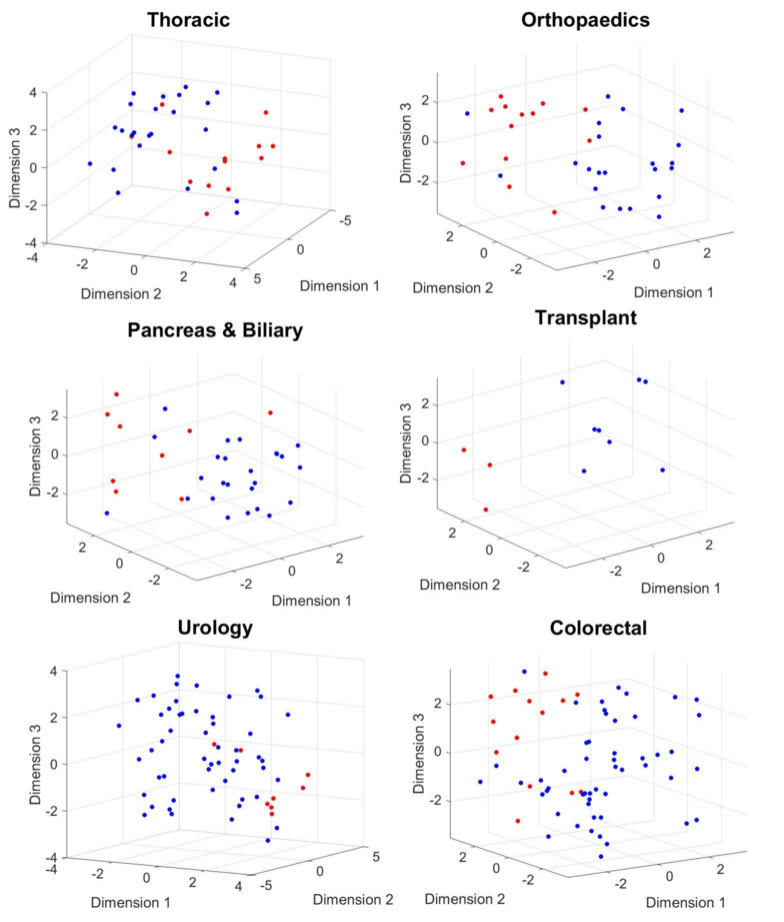
The phase information of the projected data points onto a 3D manifold extracted using the complex higher-order singular value decomposition. Mild (blue dots) versus moderate-severe (red dots) levels of pain on day 30 after surgery are considered for this plot.

**Figure 11:**
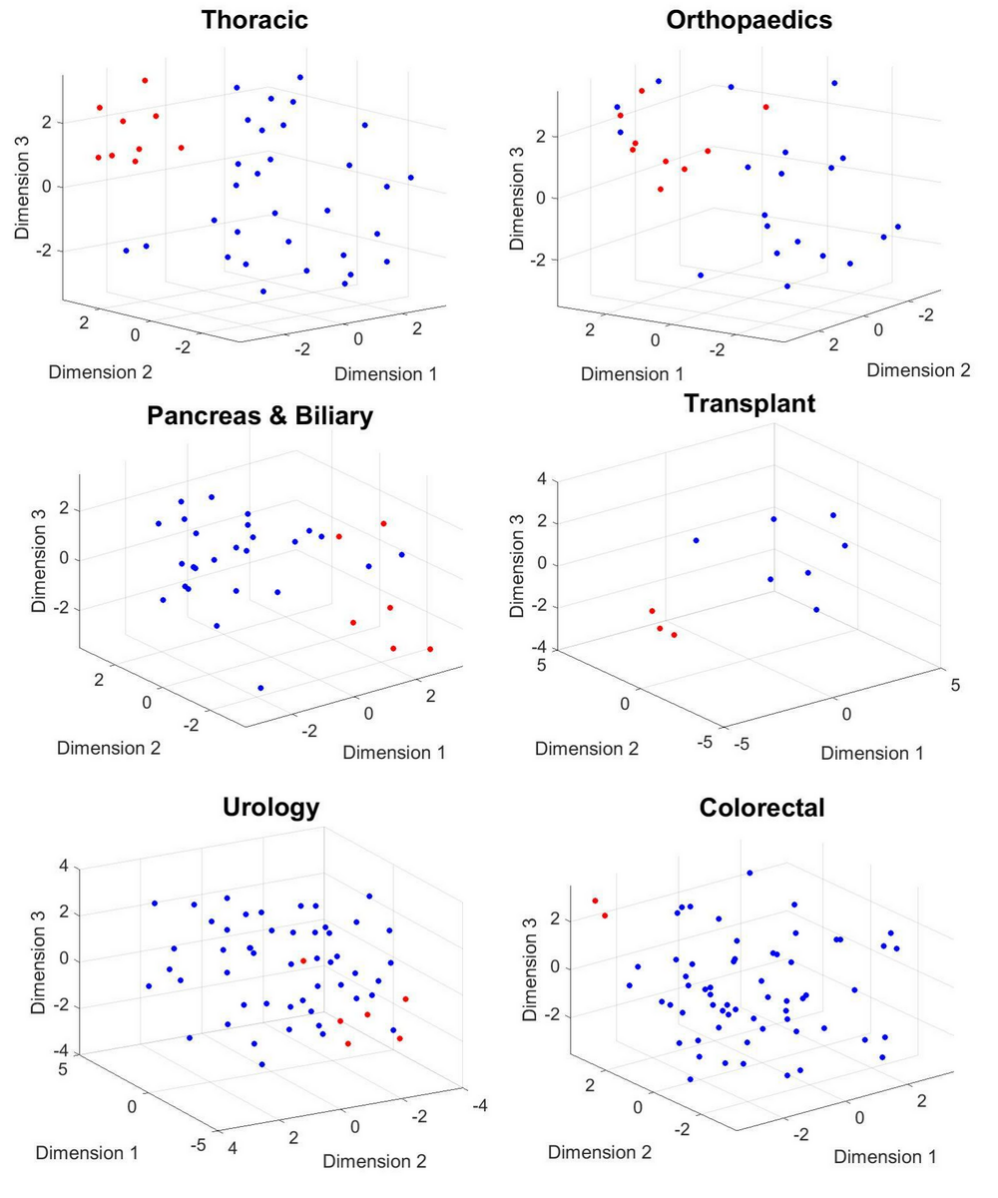
The phase information of the projected data points onto a 3D manifold extracted using the complex higher-order singular value decomposition. Mild (blue dots) versus moderate-severe (red dots) levels of pain on day 90 after surgery are considered for this plot.

We noticed that the TPR, or the PPV for the class of moderate-severe pain in the five groups related to thoracic, orthopedic, colorectal, transplant, and pancreatic and biliary surgeries, improved compared with the results of the method without rotation of physiological responses. The TPR and PPVs were 0.69 and 0.75 for thoracic surgery, 0.77 and 0.83 for orthopedic surgery, 1 and 1 for transplant surgery, 0.57 and 0.73 for colorectal surgery, and 0.67 and 0.67 for pancreatic and biliary surgery, respectively. In contrast, the PPV and TPR for the *moderate-severe* class in urological surgery remained the same. The results are summarized in Table 4. Figures 10 and 11 show the scatter plot of the phase information for patients projected onto the 3D subspace for different surgical groups. The phase information of the physiological responses of the patients in the same group of pain was more similar to each other than to those in the other groups (thoracic, orthopedic, transplant, pancreas and biliary, and colorectal surgical groups). This again emphasizes that the dynamics of patients’ physiological responses to surgical stimulation are associated with long-term POP development. We observed the same pattern in the results for postoperative day 90 (Figure 11).

**Table 4:** Performance of LDA^a^ to discriminate moderate-severe versus mild pain categories on postoperative day 30 when the physiological responses are rotated before projection. The phase information of the projected data points on a 3D manifold was used in the experiments. The patients were categorized based on their surgical services.

Surgery	Confusion matrix (patients)				Precision (PPV^b^)	Sensitivity (TPR^c^)	Specificity (TNR^d^)	AUC^e^
	TP^f^	FP^g^	FN^h^	TN^i^				
Thoracic	9	3	4	21	0.75	0.69	0.88	0.81
Orthopedics	10	2	3	20	0.83	0.77	0.91	0.87
Urology	5	2	3	50	0.71	0.63	0.96	0.87
Colorectal	8	3	6	48	0.73	0.57	0.94	0.86
Transplant	3	0	0	8	1	1	1	1
Pancreas and biliary	6	3	3	22	0.67	0.67	0.88	0.83

^a^LDA: linear discriminant analysis.

^b^PPV: positive predictive value.

^c^TPR: true positive rate.

^d^TNR: true negative rate.

^e^AUC: area under curve.

^f^TP: true positive.

^g^FP: false positive.

^h^FN: false negative.

^i^TN: true negative.

#### Results for Postoperative Day 90

We repeated the set of experiments to classify patients who reported *mild* versus *moderate-severe* levels of pain on postoperative day 90. We observed that the TPR and the PPV for the class of moderate-severe pain in the three groups related to thoracic, orthopedic, and transplant surgery increased. The results are summarized in Table 5.

The phase information of the projected data points on a 3D manifold was used in the experiments. The patients were categorized based on their surgical services.

**Table 5:** Performance of LDA^a^ to discriminate moderate-severe versus mild pain categories on postoperative day 90 when the physiological responses are rotated before projection.

Surgery	Confusion matrix (patients)				Precision (PPV^b^)	Sensitivity (TPR^c^)	Specificity (TNR^d^)	AUC^e^
	TP^f^	FP^g^	FN^h^	TN^i^				
Thoracic	8	2	1	29	0.80	0.89	0.94	0.89
Orthopedics	7	4	2	16	0.64	0.78	0.80	0.83
Urology	2	1	4	49	0.67	0.33	0.98	0.88
Colorectal	2	0	0	60	1	1	1	1
Transplant	3	0	0	7	1	1	1	1
Pancreas and biliary	4	2	2	24	0.67	0.67	0.92	0.92

^a^LDA: linear discriminant analysis.

^b^PPV: positive predictive value.

^c^TPR: true positive rate.

^d^TNR: true negative rate.

^e^AUC: area under curve.

^f^TP: true positive.

^g^FP: false positive.

^h^FN: false negative.

^i^TN: true negative.

## Discussion

### Principal Findings

This study introduced a new type of multivariate-temporal decomposition of intraoperative vital signs to explore signatures that can accurately discriminate patients who develop *mild* or *moderate-severe* pain on postoperative days 30 and 90. The method takes advantage of the fact that complex-HOSVD decomposes data into a sum of rank-1 tensors, which is a combination of modes or signatures. This method arranges the multivariate trajectory of intraoperative vital signs of various patients in a 3D data array with dimensions indexed by vital sign variable, time, and patient. This is the first time that multivariate-temporal decomposition of complex-valued intraoperative vital signs has been proposed to analyze long-term POP. Using a multivariate time structure helped us to accurately describe the dynamics of intraoperative vital signs and to find a lower-dimensional projection where differences between individual responses were encoded in the phases of complex vital signs. The primary advantage of complex HOSVD is that it discovers and examines multivariate-temporal behavior. However, complex multivariate-temporal factors are difficult to interpret as amplitude and phase relations because of Hilbert transform properties, which weigh more sudden transitions than episodes during which intraoperative vital signs change slowly. Further research is necessary to compensate for this behavior.

Although clinical verification has not yet been undertaken, this study presents a physiological interpretation of the model. This interpretation focused on the spectral dynamics of different vital signs during surgery. For the intraoperative vital signs time series used in this study, the spectral band was within the frequency band of the autonomic nervous system responding to a surgical stimulus under general anesthesia, which established the sampling rate. Variability in physiological parameters during surgery is a result of a dynamic interaction between surgery-induced perturbations in the circulatory system and the short-term compensatory response to regulate them. For instance, short-term circulation control by the baroreceptor reflex or vasomotor tone is best described by feedback models. Circulation control can be identified by a pair of input-output signals. Regarding the baroreceptor reflex, blood pressure and heart rate act as input and output signals, respectively. The transfer function parameters in the feedback system determine the input-output relation. Although the gain defines the amplitude relationship of the input-output signals, the phase determines the delay between the two. For baroreceptor reflex, the phase of the transfer function quantifies the phase shift between blood pressure and heart rate. Multivariate factors can be considered patterns of prototypical short-term circulation control in patients. Hence, the complex-valued elements of the multivariate factors may correspond to the attributes of the transfer function. In this setting, the absolute value of the elements might correlate with the gain of the transfer function, and the angle might indicate the delay between the input and output signals. For example, the strong contribution of heart rate and blood pressure and the phase shift between them in one of the extracted multivariate factors might correspond to circulation control by the baroreceptor reflex (Figure 3D). Temporal factors are highly dependent on the surgical type; therefore, they are more difficult to interpret. However, they may serve as indicators of circulation control activity during surgery.

### Conclusions

POP affects the quality of life and is associated with increased morbidity, longer recovery time, prolonged duration of opioid use, and higher health care costs. It can also lead to depression and anxiety, which can in turn worsen pain. Unfortunately, this postoperative complication remains undertreated and poorly controlled in most patients [30]. In this study, we showed that common features collected during routine anesthesia are predictive of POP-related outcomes and the development of chronic pain. The outcome of this study has potential clinical utility in using preventive treatments or starting treatment plans including medications, lifestyle changes, and therapies even before the development of moderate to severe levels of pain.

### Limitations

Our study was limited by the sampling rate of intraoperative vital signs, verbal evaluation of POP, and the small number of surgical patients involved. A higher sampling rate of vital signs would allow for a more comprehensive analysis of autonomic nervous system activity. A larger number of patients would provide valid testing of hypotheses regarding temporal and multivariate factors within surgeries. Finally, a more reliable method for assessing POP would remove noise from the data set.

This study was also limited because we considered only a small subset of relevant variables that could affect POP. In particular, we did not consider the response of patients to noxious stimuli and how it changes the effect of anesthetics and adjuvants on the dynamics of physiological parameters. This causes a failure to extract a partial correlation between the input parameters and POP.

Regarding the anesthetics delivered for the cases studied in this model, it is noteworthy that sympathomimetic agents are commonly used in anesthetics at our institution, as are both short- and long-acting beta-receptor blockers. Moreover, these agents are usually administered as bolus doses, and it remains unclear how to best model such episodic impulses into the system.

## Abbreviations

EtCO_2_: end-tidal carbon dioxide
HOSVD: higher-order singular value decomposition
LDA: linear discriminant analysis
POP: postoperative pain
PPV: positive predictive value
SpO_2_: peripheral capillary oxygen saturation
SVD: singular value decomposition
TPR: true positive rate
